# 

**Published:** 1895-04-13

**Authors:** 


**Notices of Births, Deaths, and Marriages** are inserted in this column at a charge of 2s. 6d. for an announcement not exceeding four lines.

**Notice to Advertisers and Subscribers.**

All Advertisements, Orders for Copies of The Hospital and Business Communications should be addressed to **The Manager, NOT TO THE EDITOR**, The Hospital, 428, Strand, London, W.C.

The Cost of Subscription, post free, is as follows:—Per week, 2½d.; per quarter, 2s. 8d.; per half-year, 5s. 3d.; per annum, 10s. 6d. Foreign:—Per Annum, 15s. 2d.; payable, in all cases, in advance.

### NOTICE TO CORRESPONDENTS.

All MS., Letters, Books for Review, and other matters intended for the Editor should be addressed The Editor, The Lodge, Porchester Square, London, W.

The Editor cannot undertake to return rejected MS., even when accompanied by stamped directed envelope.

**"Burdett's Hospital Annual," 1895,**

Ready Immediately.

Sixth year of publication. About 1000 pp. Scarlet cloth, gilt lettered. Price 5s. A most complete guide to British, Colonial, Indian, and American Hospitals, Dispensaries, Nursing and Convalescent Institutions, and Asylums. A few copies of the "Annual" for 1894 may still be obtained on application to the Publishers, The Scientific Press (Limited), 428, Strand, London, W.C.

**NOTICE.—The Index for Vol. XVII. (ending March 30th, 1895) is now on sale at the Office of this Journal. Price 2½d., post free.**

### Scraps and Gleanings.

The Drapers' Company has contributed 100 guineas towards the special fund of St. Thomas's Hospital.

The secretary of the Swindon Victoria Hospital is urgently pleading for help towards the funds of that institution.

The members of the Halifax Provident Dispensary (which is now carrying on a gratifying work) have increased to 400.

An in-patients' department is going to be opened at the Birmingham Ear and Throat Hospital. This is to accommodate ten beds.

The twenty-third anniversary festival of the Provident Surgical Appliance Society is to be held on May 13th at the Hotel Metropole, Charing Cross.

By the will of the late Mayor of Kendal, Mr. Alderman W. Bindloss, amongst many munificent bequests, to local and other charities, £1,000 each is left to the Middlesex and St. George's Hospitals.

The London and South-Western Railway Company have contributed the sum of £105 to the St. Thomas's Hospital Special Appeal Fund, and have increased their annual subscription from £15 to £26 5s.

Forty-four thousand pounds have been left by M. Cohen, a diamond merchant of Paris, for the erection of a hospital for sick children to contain a hundred beds. It is to be situated in the suburbs of Paris.

Designs for the new branch building of the Birmingham General Dispensary at Smallheath have been accepted from Mr. Doubleday, and the work of construction will be commenced as soon as the weather permits.

The committee of the Nottingham and Notts Convalescent Homes propose expending £530 on the purchase of an additional cottage property to be used as an extension, in the form of a children's home, to the already existing buildings.

Statistics show the last year to have been one of prevalent epidemics in Coventry. At the City Hospital six different varieties of infectious disease have been treated, and the provision of a separate small-pox hospital is urgently advocated.

The twenty-first annual meeting of the Hospital Saturday Fund was lately held at the Mansion House. The committee stated in their report that the total amount awarded to the various medical charities is £17,608, being slightly below that which was awarded last year.

Owing to the distress which has lately prevailed in Southampton, the Hospital Sunday Fund Committee have decided to postpone the general annual collections till about the second week in May. In several places of worship, however, collections have already been made on behalf of the fund.

The new Smallwood Hospital at Redditch, named after its founders, the late Edwin Smallwood and Mr. William Smallwood, his brother, has been completed at a total cost for building, site, and furniture, of about £10,000. The number of beds is 28. The hospital is to be opened on May 1st.

The Princess of Wales has sent, in aid of the children attending the central dining-hall of the Board School Children's Free Dinner Fund (at Omega Hall, St. John's Wood) a handsome present of fish. On the 22nd inst. H.R.H. the Duchess of York signified her intention of sending a similar gift.

A satisfactory report is presented this year of the Cirencester Cottage Hospital, and its funds.

The packing-houses of Messrs. Armour, the purveyors of preserved meats in Chicago, are one of the sights of that city. They are scrupulously clean, and open to the public, and therefore the censure conveyed in a recent article in the *Times* on Chicago packing-houses cannot be applied to them.

The managing staff of the Stanley Hospital, Liverpool, are earnestly appealing for funds, the expenditure of the year having raised the indebtedness of the hospital to £1,137; and it has been pointed out that the deficit balance is increasing every year, and further financial support is very earnestly needed.

At the annual meeting of the City of London and East London Dispensary, a satisfactory financial report was presented. Outstanding liabilities, it was stated, had all been discharged, and a sum of £1,400 in Consols stood to the credit of the institution. Receipts from subscribing members had also increased.

The committee of the Royal Surrey County Hospital have the gratification of stating that the improvement in the financial position anticipated in the previous report is an accomplished fact. In addition to £500 offered to the hospital from a friend of, and through, Mr. Brodrick, the Bishop of Winchester has contributed £25.

A local benefactor has offered to provide the labour of certain improvements to the walls of the Central Provident Dispensary at Bedford, if the committee pay for such material as is required. This dispensary is increasing the sphere of its usefulness; and members' subscriptions are steadily on the increase.

The number of patients at the Westminster Ophthalmic Hospital has far exceeded the record of any previous year. The necessity for a new building on a larger site is now being forced upon the committee, and special funds will forthwith be asked for from the public. A bazaar is to be held in May to raise money for this object.

The "Annales de l'Institut Pasteur" gives the number of patients under treatment there during the last three months of 1894 as 333. In 316 of these cases the bites were inflicted by dogs, the other 17 by cats; 58 of the patients were bitten by animals experimentally proved to be mad, 210 by animals declared to be mad by veterinary certificate, and in 6 suspicion only existed.

The Devon and Exeter Hospital has lately come in for a stroke of good fortune. A sum of £1,000 was left to the institution by the late Miss Hodges, of Cullompton, under the provision that the hospital should "also occupy the position of residuary legatee under her will." It is now stated that the hospital will altogether benefit by Miss Hodges' generosity to the amount of some £5,000. The authorities are much to be congratulated.

The first annual meeting of the Glasgow Hospital Saturday Fund was held on March 20th, the Lord Provost (Mr. James Bell) presiding. The committee acknowledged with gratitude the hearty way in which churches and Sunday schools of all denominations had fallen in with the scheme. The total amount then accruing to the Glasgow charities during this first year reached the by no means inconsiderable sum of £5,208 13s. 11d. The new organisation had resulted in a considerable increase to the funds of the hospital.

### Books Received.

Swan Sonnenschein.

"A Student's Text-book of Botany." Sydney H. Vines, M.A.

A. D. Innes and Co.

"Thirteen Doctors." Mrs. J. K. Spencer.

Charles Griffin and Co.

"A Handbook of Hygiene." A. M. Davies, M.R.C.S.

"Infancy and Infant Rearing." John Benjamin Hellier.

Macmillan and Co.

"Text-book of Anatomy and Physiology for Nurses." Diana Clifford Kimber.

**Periodicals and Pamphlets.**—English Illustrated, Sunday at Home, Leisure Hour, Penny Tales for the People, Boy's Own Paper, Girl's Own Paper, Indian Medico-Chirurgical Review, The Nursing World, Popular Science News, Cornhill Magazine, Humanitarian, Cassell's Family Magazine, The Therapist, Dissections Illustrated, The Corpuscle, The Practitioner.

### Medical and Administrative Appointments.

THE HOSPITAL FOR SICK CHILDREN,

